# Neonicotinoid pesticides exert metabolic effects on avian pollinators

**DOI:** 10.1038/s41598-021-82470-3

**Published:** 2021-02-03

**Authors:** Simon G. English, Natalia I. Sandoval-Herrera, Christine A. Bishop, Melissa Cartwright, France Maisonneuve, John E. Elliott, Kenneth C. Welch

**Affiliations:** 1 Department of Cell and Systems Biology, University of Toronto, Toronto, ON Canada; 2 Department of Biological Sciences, University of Toronto Scarborough, Toronto, ON Canada; 3 Department of Ecology and Evolutionary Biology, University of Toronto Scarborough, Toronto, ON Canada; 4 Wildlife Research Division, Environment and Climate Change Canada, Delta, BC Canada; 5 Ecotoxicology and Wildlife Health Division, Environment and Climate Change Canada, Ottawa, ON Canada; 6 Centre for the Neurobiology of Stress, University of Toronto Scarborough, Toronto, ON Canada

**Keywords:** Animal physiology, Homeostasis, Ecophysiology

## Abstract

Neonicotinoids are neurotoxic systemic insecticides applied extensively worldwide. The impacts of common neonicotinoids like imidacloprid on non-target invertebrate pollinators have been widely studied, however effects on vertebrate pollinators have received little attention. Here, we describe the first study evaluating the effects of short-term (3 d) exposure to a range of environmentally relevant concentrations (${0.2}\,\upmu \hbox {g g}^{-1}$ to ${2.5}\,\upmu \hbox {g g}^{-1}\cdot$Body Weight) of imidacloprid on wild-caught ruby-throated hummingbirds. Within 2 h of exposure, hummingbirds exhibited a significant depression in energy expenditure (up to $25\% \pm 11\%$). We did not observe significant effects on foraging behaviour measured in the subsequent 2 h to 4 h, although the effect size estimate was large (0.29). We also analyzed tissues collected 24 h after the final dose and did not observe significant effects on immune response or cholinesterase activity, although this may be related to our small sample size. We determined that hummingbirds excrete imidacloprid quickly (elimination half-life of $2.1\hbox { h} \pm 0.1\hbox { h}$) relative to other bird species. Hummingbirds have high energetic demands and store relatively little energy, especially during migration and breeding seasons. Therefore, changes in their metabolism following exposures to imidacloprid observed herein could bear important survivorship consequences for hummingbirds.

## Introduction

Neonicotinoids are now the most widely used class of insecticides in the global market^1,2^. International neonicotinoid sales are in excess of $3.5 billion, nearly 85 % of which is accounted for by imidacloprid, thiamethoxam, and clothianidin^3^. These systemic chemicals are translocated to all tissues of treated plants, putting a wide range of invertebrate and vertebrate pollinators at risk of exposure^4–8^. Furthermore, neonicotinoids spread rapidly through the environment in runoff due to their relatively high water solubility and their persistence in plants and soil^9–11^. The persistent and systemic nature of neonicotinoids causes exposure to wildlife via multiple routes including plant tissues (pollen, nectar, seeds)^7,12^, contaminated water^7,13,14^, and/or dust/wind^15,16^. Concern over the impact of neonicotinoids on non-target animals has spurred toxicity studies, particularly on invertebrate pollinators^17–20^, however little attention has been paid to essential vertebrate pollinators, including bats and birds^21,22^. We therefore investigated the effects of neonicotinoids on hummingbirds, key pollinators in the Americas^23^.

Hummingbirds present life history traits that make them especially vulnerable to pesticide exposure and its deleterious effects. First, they can feed on thousands of flowers per day, potentially consuming large amounts of contaminated nectar and pollen. Furthermore, many species perform long migrations^24–26^ across regions with different regulations on pesticide use, which could increase the risk of exposure to these substances along their journey. Lastly, their small body sizes and high metabolic rates might exacerbate the potential deleterious effects of toxicants like neonicotinoids^27^. Exposure in hummingbirds can occur via direct contact through their skin, consumption of contaminated nectar and pollen, or consumption of poisoned invertebrates^10,28–30^. Multiple neonicotinoids, including imidacloprid, and related butenolide compounds have been detected in cloacal fluid of the rufous hummingbird (*Selasphorus rufus*), a declining species in North America, substantiating concerns of widespread, chronic, non-target exposure^31^. While effects on granivorous birds have been observed^32–34^, and declines in insectivorous birds populations have been correlated to neonicotinoid concentrations in the environment^35^, effects on nectarivorous birds such as hummingbirds have yet to be investigated. Therefore, we assessed the effects of imidacloprid exposure on a suite of metrics in the ruby-throated hummingbird (*Archilochus colubris*), a common species in Eastern North America.

Body weight measurements have been used to detect metabolic effects of neonicotinoid exposure in grassland birds^32^, however, body weight as a proxy for energy balance in hummingbirds presents greater challenges. This family of small, energetically extreme animals regularly experience relatively large variations in body weight throughout the day as they feed, urinate, and expend energy. Hummingbirds can lose up to 10% of their mass overnight^36^. Therefore, we applied respirometry techniques and predicted that exposure to imidacloprid would induce a dose-dependent decrease in energy expenditure and subsequently, feeding and flying behaviour^32,33,37^. Our behavioural assay simulated a swaying flower like that which a hummingbird would encounter while foraging in the wild. We used heterophil/lymphocyte ratios as a biomarker of stress-induced suppression of the humoral immune response for its advantages as a low cost, low sample volume, and relatively time-insensitive technique for measuring stress in birds^38^. We predicted that birds would exhibit an increase in heterophil/lymphocyte ratios, a result that has been observed in vertebrates exposed to neonicotinoids^39^. We also performed cholinesterase activity assays to test for effects of short-term, sub-lethal imidacloprid exposure on the cholinergic system of hummingbirds. Data surrounding the responses of the cholinergic system to neonicotinoids is equivocal, where responses are dose, taxa, tissue, and compound specific^34,40–44^. Finally, we collected cloacal fluid following dosage to quantify the elimination rate of unmetabolized imidacloprid to gain insight into exposure levels experienced by wild hummingbirds. Our study highlights the need for evidence-based regulations on the use of neonicotinoids, considering the growing body of research demonstrating effects on both vertebrates and invertebrates.

## Results

Normalized average energy expenditure measured using respirometry followed a dose-response curve where average energy expenditure decreased by up to $25\% \pm 11\%$ in the high dose group within 2 h after dosage. There were no significant effects on behaviour in the 4 h to 6 h time period after dosage. The effect size estimate for change in time spent foraging during this period was large ($p =0.06$; effect size: 0.29; 95% CI − 0.23 to 0.60). No significant differences in heterophil/lymphocyte ratios were observed among treatment groups. Cholinesterase activity showed no significant changes in either brain or flight muscle tissues. Imidacloprid was excreted according to a first-order kinetics model, with an elimination half-life of 2.1 h ± 0.1 h.

### Metabolism

**Figure 1:**
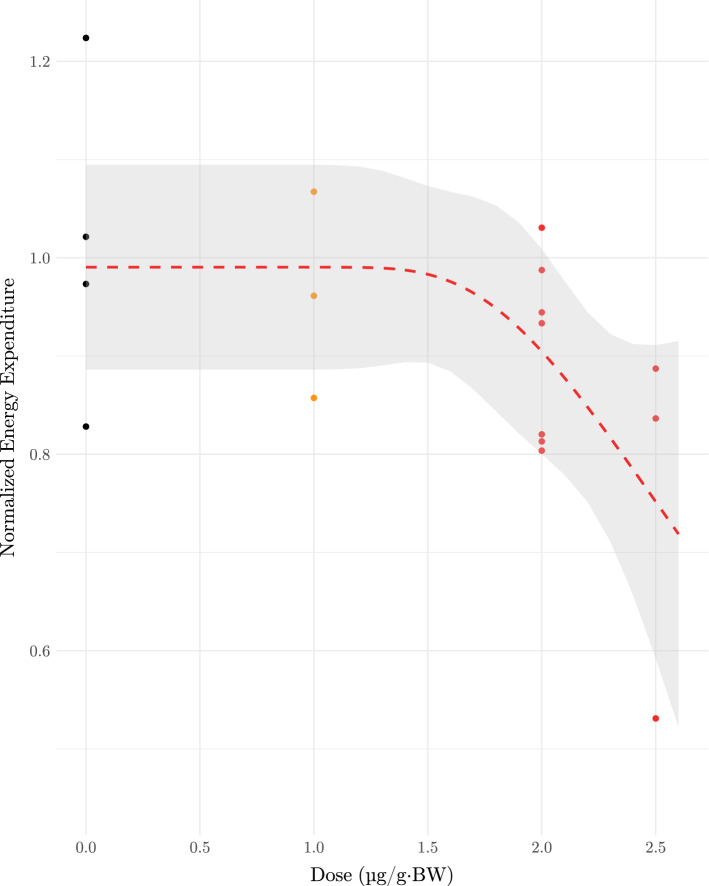
Energy expenditure of ruby-throated hummingbirds 1.5 h to 2 h after dosage with ${0.0}\,\upmu \hbox {g g}^{-1}$ to ${2.5}\,\upmu \hbox {g g}^{-1}\cdot$Body Weight (BW) imidacloprid follows a Weibull type 2 dose-response curve. Birds in the control group increased energy expenditure by an average of ${1\,\% \pm 7\,\%}$ on days following dosage with the control solution, while birds in ${1.0}\,\upmu \hbox {g g}^{-1}\cdot$BW, ${2.0}\,\upmu \hbox {g g}^{-1}\cdot$BW, and ${2.5}\,\upmu \hbox {g g}^{-1}\cdot$BW groups reduced energy expenditure on dosing days by an average of ${6\,\% \pm 5\,\%}$, ${10\,\% \pm 3\,\%}$, and $25\% \pm 11\%$ respectively. Shaded region indicates the 95 % confidence band, estimated from fitted values.

Exposure to imidacloprid caused a significant reduction in mean energy expenditure in birds along a sigmoidal dose-response curve between 1.5 h to 2 h after dosage ($p = 0.028$) (Fig. 1). Birds in the control group increased energy expenditure by an average of ${1\,\% \pm 7\,\%}$ on dosing days, while in the 1.0 $\upmu \hbox {g g}^{-1}\cdot$BW group, birds reduced energy expenditure by an average of ${6\,\% \pm 5\,\%}$. In the 2.0 $\upmu \hbox {g g}^{-1}\cdot$BW group, birds reduced energy expenditure on dosing days by an average of ${10\,\% \pm 3\,\%}$. Finally, birds in the ${2.5}\,\upmu \hbox {g g}^{-1}\cdot$BW group reduced energy expenditure on dosing days by an average of $25\% \pm 11\%$ (Fig. 1).

### Flying, and feeding behaviour

No significant effects were observed in the change in total foraging ($p =0.06$; effect size: 0.29; 95% CI -0.23 to 0.60) or non-foraging ($p = 0.85$; effect size: − 0.16; 95% CI − 0.23 to 0.09) flight time between pre-dose and post-dose conditions among dosing groups. No effect was observed in average instances of flight normalized to pre-dose averages, whether foraging flights ($p = 0.71$) or not ($p = 0.83$). We did not observe a significant effect in the amount of maintenance diet consumed among dosing groups on post-dose days as compared to pre-dose averages ($p = 0.55$).

### Stress induced suppression of the humoral immune response

No significant effect of short-term dosing was observed on heterophil/lymphocyte ratios ($p=0.98$; effect size: − 0.19; 95% CI − 0.20 to 0.20). Mean heterophil/lymphocyte ratios of 2 independent counts were taken for each individual.

### Neurotoxicity

No significant effect of imidacloprid dosing was observed on the specific activity of cholinesterase in brain tissue ($p=0.16$; effect size: 0.11; 95% CI -0.17 to 0.36) or muscle tissue ($p=0.21$; effect size: 0.08; 95% CI -0.17 to 0.33).

### Imidacloprid clearance and toxicokinetics

**Figure 2:**
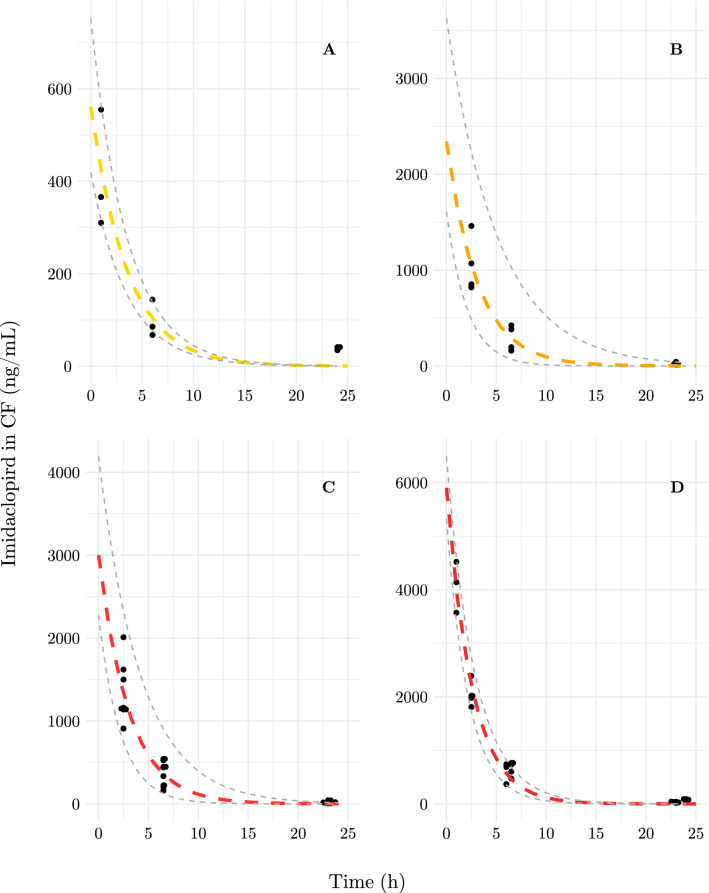
Excretion model of imidacloprid in the cloacal fluid of ruby-throated hummingbrids exposed to (**A**) ${0.2}\,\upmu \hbox {g g}^{-1}\cdot$Body Weight (BW); (**B**) ${1.0}\,\upmu \hbox {g g}^{-1}\cdot$BW; (**C**) ${2.0}\,\upmu \hbox {g g}^{-1}\cdot$BW; or (**D**) ${2.5}\,\upmu \hbox {g g}^{-1}\cdot$BW. Convergence on parameter estimates was achieved by nonlinear least-squares regression. 95 % confidence intervals for excretion models described by Eq. (3) were determined by bootstrap resampling^45^. Parameter estimates and confidence intervals are listed in Table 1. Samples were pooled by time point across dosing days by individual. Summary statistics of analytical chemistry results are reported in Table S2.

24 h after exposure, hummingbirds cleared ${97.5\,\% \pm 0.6\,\%}$ of the imidacloprid doses. All birds in the control group and the pre-dose condition showed either non-detectable ($n = 7$) or only trace ($n = 1$; less than ${1}\,\hbox {ng}\hbox { mL}^{-1}$ at each time point) levels of imidacloprid in cloacal fluid. Control birds in the post-dose condition also exhibited either non-detectable or trace levels of imidacloprid in cloacal fluid, validating that birds did not experience consequential levels of incidental exposure (Table S2). The first-order excretion models (Fig. 2) were fitted based on concentrations of imidacloprid in cloacal fluid samples collected from ruby-throated hummingbirds, quantified by LCMS. Excretion models are presented for all dosing levels (Fig. 2) where the excretion rate constant (*k*) in the first-order model is expected to be dose-independent, while the excretion coefficient (*a*) is dose-dependent.

**Table 1:** Parameter estimates for first-order toxicokinetics excretion model (3) by imidacloprid dose ${0.2}\,\upmu \hbox {g g}^{-1}$ to ${2.5}\,\upmu \hbox {g g}^{-1}\cdot$Body Weight (BW). Parameter *k* is the excretion rate constant and parameter *a* is the dose-dependent excretion coefficient.

Dose ($$\upmu \hbox {g g}^{-1}$$$$\cdot$$BW)	Rate constant (*-k*)			Excretion coefficient (*a*)		
	95% CI			95% CI		
	Estimate	Lower	Upper	Estimate	Lower	Upper
0.2	− 0.282	− 0.522	− 0.162	543.8	419.1	752.6
1.0	− 0.321	− 0.479	− 0.194	2341.5	1611.1	3627.2
2.0	− 0.326	− 0.446	− 0.236	3001.6	2279.9	4194.5
2.5	− 0.392	− 0.451	− 0.345	5903.0	5328.6	6494.0

Evaluating the mean elimination half-life, $t_{1/2}$, from the rate constant estimates in table 1 using Eq. (4), we determined that unmetabolized imidacloprid has a half-life of $2.1\hbox { h} \pm 0.1\hbox { h}$ in cloacal fluid of ruby-throated hummingbirds.

## Discussion

Validating our prediction, metabolic rate measurements on hummingbirds demonstrated that within 2 h of exposure, birds reduced their average energy expenditure in a dose-dependent manner (Fig. 1). Exposure to sub-lethal levels of imidacloprid reduced foraging efficiency among invertebrate pollinators^46^, though until now, there was no precedent for similar effects in avian pollinators. A reduction in energy expenditure (Fig. 1), even temporarily, could have severe implications for animals like hummingbirds which have high energy demands, particularly during the breeding season when critical and energetically costly behaviours including courtship and territorial defense occur frequently^24,30,47^. Furthermore, hummingbirds are chronically exposed to imidacloprid and other systemic insecticides in the wild^31^, therefore repeated or ongoing reduction in energy expenditure could ensue.

**Figure 3:**
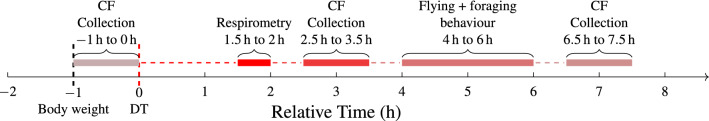
Daily experimental timeline for days 1 through 6 of trials where on days 1 through 3, a control solution (20% w/v sucrose solution) is given in all groups and on days 4 through 6, dosing solutions were administered. Times of data collection are shown relative to Dose Time (DT). Terminal tissue sampling occurred on day 7 at DT, 24 h after the final dose was administered.

Changes to energy expenditure induced by neonicotinoids may impair ecologically important behaviours in birds. In songbirds, food consumption was impacted by imidacloprid exposure at similar relative doses to those used in this study (10% of the LD50 reported for a metabolically comparable bird), though this effect abated following 3 d and 14 d recovery periods^32^. Contrary to our prediction, we did not observe changes in total consumption of the maintenance diet or flying activity (Fig. S1), however the estimated effect size was large among foraging flights of imidacloprid dosed birds. In addition to our small sample size, high inter-individual variation in the behaviours evaluated may limit our ability to detect subtle effects of exposure with statistical significance^48^. Due to logistical constraints, we could not simultaneously assay foraging behaviours while birds were in respirometry chambers 1 h to 2 h after dosing (Fig. 3). As indicated by our toxicokinetic models (Fig. 2) and time-dependent effects reported in studies with migratory songbirds^32^, this may have been the key window during which acute effects on behaviour were strongest. Therefore, the non-significant yet large effect size may be related to an attenuation of the toxic effects of dosage over time. This could be attributed to mechanisms including, A) the rapid metabolism and clearance of the pesticide (Fig. 2), or B) the higher toxicity of metabolites compared to unmetabolized imidacloprid, which peak in plasma after parent imidacloprid in quail, and then are cleared rapidly (2.2 h)^49,50^. Therefore, chronic exposure to imidacloprid in the wild^31^ may induce sub-lethal, yet deleterious behavioural effects on hummingbirds.

On a tissue basis, exposure to environmental toxicants can increase baseline levels of stress hormones in blood, which in turn suppress the humoral immune response, and thus the capacity of birds to mount an immune response against novel antigens^34,51,52^. The rise of bacterial diseases in birds over the past two decades has been speculatively attributed to a widespread suppression of immunity related to pesticide exposure in birds^53^. We did not find support for this in our 3 d short-term dosing study using heterophil/lymphocyte ratios as a biomarker for stress induced suppression of the humoral immune response (Fig. S2). Studies using techniques which require additional animal handling have identified cell-mediated immune suppressive effects of neonicotinoid exposure on birds^34^. This immune suppression in vertebrates is thought to be related to elevated levels of stress hormones including corticosterone^39^. Future studies should include periods of more than 3 d between treatment with neonicotinoids and tissue collection to allow for adequate turnover of lymphocytes^54^. It has also been suggested that multiple biomarkers of physiological stress must be considered concurrently, including basal corticosterone levels^55^. Individually, these assays may not be accurate reflections of the stress-induced suppression of the humoral immune response.

On a biochemical level, our investigation into the neurotoxic effects of short-term imidacloprid exposure yielded results contradictory to our predictions. Neurotoxic effects in non-target organisms have been documented, although reliable biomarkers are elusive^6,32,33,50^. Toxicological studies using cholinesterase activity as a marker of imidacloprid exposure in birds have yielded results of both decreased^34^ and increased^43^ activity. Following 3 d acute dosing, we observed no significant variation in cholinesterase activity in hummingbird brain (Fig. S3) or muscle (Fig. S4), and only a moderate effect size in both tissues related to imidacloprid dosing. Therefore, a repeated, systemic exposure regime may have induced a continuous, or additive, imbalance in neurotransmission, causing the cell to activate compensatory mechanisms such as the downregulation of cholinesterases or alteration of cholinesterase activity^40^. Thus, chronic or long-term exposure may have neurotoxic effects quantifiable through the use of cholinesterase activity assays in hummingbirds. However, based on rapid clearance (Fig. 2) and recovery from short-term dosing^32^, an alternative sampling or dosing regime is needed for future investigations.

In addition to these endpoints, we contextualized our lab-based dosing study by measuring how administered doses are eliminated over a 24 h period, thus providing insight into the level of exposure experienced by wild hummingbirds. Excretion of unmetabolized imidacloprid in cloacal fluid followed a first-order kinetics model (Table 1). Hummingbirds are exceptionally efficient at concentrating solutes and rapidly eliminating excess water^56^ and have rapid energy turnover rates^57^. These traits introduce a unique temporal consideration for neonicotinoid elimination in hummingbirds. The elimination half-life for imidacloprid in hummingbirds ($2.1\hbox { h} \pm 0.1\hbox { h}$) is approximately 67% of the elimination half-life reported in plasma of quail (*Coturnix japonica*) administered a single dose of imidacloprid^49^. This is likely due to the rapid glomerular filtration rates in hummingbirds and inverse relationship describing urine-concentrating abilities and animal size^58,59^. Notably, several metabolites of imidacloprid have higher toxicity than the unmetabolized compound, and in quail (*Coturnix japonica*) plasma concentration peaks approximately 1 h after dosage with imidacloprid^49,50,60^. Our data (Fig. 2,3) suggest that toxic effects in hummingbirds (Fig. 1) may overlap with this peak in metabolite concentration reported in quail^49^. Recovery from these effects soon after exposure may have precluded our ability to detect significant effects on other measures collected later in the experimental period.

The environmentally relevant concentrations of imidacloprid used in this study span the range of exposure birds face drinking from treated flowers in North America^10,31^ to high exposure of 10 % of the LD50 in canaries^61^, a sub-lethal concentration expected to produce toxic effects^32^. Our most critical finding considering the physiology of hummingbirds was an immediate depression of energy expenditure as a result of imidacloprid exposure for just 3 d. This could have considerable impacts on foraging, migration, and breeding success in hummingbirds following acute and chronic exposure to neonicotinoids. Although we did not observe significant effects on the tested feeding and flying behaviours (Fig. 3), the effect size estimate for the change in time spent in foraging flights was large and followed a similar trend to energy expenditure measured sooner after dosage. There were no significant effects on stress-induced immune response, or cholinesterase activity, although these negative results may be related to our small sample size and time of tissue collection relative to dosing. Our toxicokinetics results demonstrate that imidacloprid in ruby-throated hummingbirds is excreted according to a first-order model, wherein a constant proportion of the compound is eliminated after each half-life of approximately 2 h. When considered with the former suite of assays, these data suggest that as imidacloprid is excreted and exposure declines, effects may also diminish; when exposed chronically however, wild hummingbirds may not experience this attenuation in negative effects. Chronic neonicotinoid exposure has been confirmed for wild hummingbirds in western North America^10,31^ and is consisten with reports of dt50 in media... reports on the dt50 but is likely widespread given that reports on the time for 50% dissipation ($\hbox {DT}_{{50}}$) of imidacloprid in the field suggest a $\hbox {DT}_{{50}}$ upward of 3 years in some media^17, 62^. Our data indicate that energy expenditure is the most sensitive endpoint in ruby-throated hummingbirds following short-term imidacloprid exposure.

## Materials and methods

All experimental procedures were approved by the University of Toronto animal care committee (Animal Use Protocol number 20012112) and conformed to guidelines prescribed by the Canadian Council on Animal Care.

### Animal capture and husbandry

Wild male ruby-throated hummingbirds (*Archilochus colubris*; $n = 23$; mass range during experimental period: 2.59 g to 4.52 g), were caught on the University of Toronto Scarborough Campus (${43.7838}^{\circ }\hbox {N}$, ${79.1875}^{\circ }\hbox {W}$) or the University of Western Ontario campus (${43.0096}^{\circ }\hbox {N}$, ${81.2737}^{\circ }\hbox {W}$) using box traps modified with hook-and-loop fastener tape on a drop door containing hummingbird feeders. Birds were trapped between 06:00 h and 12:00 h during the months of May through September of 2017, 2018, and 2019. Pilot trials were conducted in 03/2018. Birds in the pilot study were on wintering/migratory seasonality with a 12 h daylight schedule. Subsequent trials were conducted between 04/2019 and 01/2020. In 04/2019, birds were under breeding seasonality (14 h daylight) and in 01/2020, birds were under wintering/migratory seasonality (12 h daylight) during experimental trials. The daylight schedule approximated the photoperiod encountered as part of annual migrations to Central America and back. Upon capture, hummingbirds were quickly transported to metal EuroCages (${50.8 \times 91.5 \times 53.7}\,\hbox {cm}$ ($\hbox {L}\times \hbox {W}\times \hbox {H}$)) at the animal care facility where they were housed individually and acclimated to feed from syringe feeders. Birds were provided an 18 % (w/v) Nektar Plus (Guenter Enderle, Tarpon Springs, FL, USA) solution (henceforth referred to as maintenance diet), which was consumed ad libitum, and syringes were replaced daily (range of average consumption of daily maintenance diet during study period was 5.4 mL13.2 mL).

### Experimental design

Birds drank solutions of imidacloprid (IMI; Sigma-Aldrich Cat. No. 37894) dissolved in a 20% w/v sucrose solution and were randomly assigned to either control (${0.0}\,\upmu \hbox {g g}^{-1}\cdot$BW), low (${1.0}\upmu \hbox {g g}^{-1}$), middle (${2.0}\upmu \hbox {g g}^{-1}$), or high dose (${2.5}\upmu \hbox {g g}^{-1}$) groups (n = 7, 4, 8, 4, respectively). Stock solution concentrations were analytically confirmed (low: ${0.32}\,\hbox {gL}^{-1}$, middle: ${0.59}\,\hbox {gL}^{-1}$, high: ${0.78}\,\hbox {gL}^{-1}$) such that a 3 g bird dosed with ${10}\,\upmu \hbox {L}$ of solution would receive the dosage rate corresponding to either the low, middle or high dose. The volume of imidacloprid stock solution used for dosage was adjusted on a body weight (BW) basis, pipetting from the stock solution into a new nectar syringe and drawing up to a final volume of ${50}\,\upmu \hbox {L}$ with 20% w/v sucrose solution, ensuring that birds received the same dosage rate throughout the trial. Birds were deprived of their regular nectar solution for 10 min to 15 min consumed the entire small-volume dosing solution within 10 min of being offered the solution. The dose was considered to be delivered when there was no visible solution remaining in the transparent syringe.

Doses were established within a range spanning expected exposure in a bird drinking ${10}\,\hbox {mLd}^{-1}$ from contaminated flowers^10^ up to 10 % of the LD50 in canaries^61^ (*Serinus canaria*, LD50: ${25}\,\upmu \hbox {g g}^{-1}$ to 50 $\upmu \hbox {g g}^{-1}$), similarly small birds with fast metabolic rates to target a sub-lethal concentration expected to produce toxic effects^32^. When energy demands are high, hummingbirds may consume over three times their body weight in nectar^63^, therefore ${10}\,\hbox {mLd}^{-1}$ is a probable figure for contaminated nectar consumption. Pooled blueberry flower samples collected about 1 year after treatment with imidacloprid contained the neonicotinoids at a concentration of ${5.16}\,\hbox {ng g}^{-1}$^10^. We extrapolated our very low and low dose concentrations based on these data. We stipulate that given the flower sample is a pooled sample, it was collected from flowers long after treatment, and there are different regulations on pesticide use within the ruby-throated hummingbird’s range, these doses were environmentally relevant.

We tested multiple intermediate doses which allowed us to explore dose-response relationships in observed effects^64^. Pilot experiment data with control, very low (${0.2}\,\upmu \hbox {g g}^{-1}$), or high dose (${2.5}\,\upmu \hbox {g g}^{-1}$) (n = 3 per group) are included for cholinesterase activity and toxicokinetic elimination analyses. Other metrics including behaviour and energy expenditure were not included from the pilot study due to differences in data collection protocols and are not strictly comparable. Behavioural data collection, cloacal fluid (CF) collection, and respirometry occurred over 6 days, where pre-dose data were collected for each animal on days 1 through 3, and dosing occurred once per day at 11:00 on days 4 through 6. Body weight measurements were taken daily at 10:00. The body weights of birds on the first day of experimentation ranged from 2.70 g to 4.52 g. For simplicity, 11:00 on days 1-6 is referred to as Dose Time (DT). Terminal sampling and tissue collection occurred 24 h after the third dose was administered. Birds were sacrificed by decapitation following isoflurane overdose, and whole blood, flight muscle, liver, brain, and heart tissues were rapidly excised, flash frozen in liquid nitrogen, and stored at ${-80}\,^{\circ }\hbox {C}$ until downstream analysis, except in the case of blood which was immediately used for blood smear preparation.

### Respirometry

Oxygen consumption and carbon dioxide production rates were measured using open-flow chamber respirometry^65^. Airflow through three metabolic chambers and one empty reference chamber was maintained at a rate of ${300}\,\hbox {mL min}^{-1}$. Excurrent air from the chambers was sub-sampled at ${100}\,\hbox {mL min}^{-1}$ sequentially starting with the reference chamber at using a Turbofox-5 (Sable Systems International Las Vegas, NV, USA). Sub-sampled air was passed through a water vapour pressure analyzer, a drying column (Indicating Drierite, W.A. Hammond Drierite, Xenia, Ohio, USA), carbon dioxide meter, and finally an oxygen analyzer (Turbofox-5, Sable Systems International). The oxygen and carbon dioxide analyzers were calibrated according to manufacturer instructions using well-mixed ambient air for the oxygen analyzer, and zero and $0.25 \,\%\hbox {CO}_{2}$ reference gases for the $\hbox {CO}_2$ analyzer. Respirometry data were recorded at a frequency of 1 Hz using Expedata software (v. 1.84, Sable Systems) for 5 min while sampling from the empty reference chamber, followed by three 7 min recording periods from each of the chambers holding a bird. After this 26 min period, sub-sampling was resumed from the reference chamber for another 5 min followed by another 7 min sub-sampling period from experimental chambers. A final 5 min sampling of the reference chamber concluded the respirometry data collection, and birds were returned to the cloacal fluid collection chambers approximately 60 min after initially being placed in respirometry chambers.

### Behavioural data collection and processing

Video recordings of birds were collected for 2 h, starting 4 h after DT (15:00–17:00). At the start of the recording period, birds were returned to their home cages where they could feed ad libitum by hovering and tracking a syringe on a 10 cm arm oscillating through a ${90}^{\circ }$ range along a lateral arc at a speed of 15 RPM. Video recordings were analyzed for time spent in flight, subdivided into foraging and non-foraging flights. Foraging flights were defined as flights where the bird contacted the hover feeder with their bill. Total consumption of the maintenance diet over this 2 h period was recorded.

### Heterophil/lymphocyte ratios

Approximately ${2}\,\upmu \hbox {L}$ of blood was collected for blood smear preparation immediately following sacrifice. After smearing, slides were left to air dry for a minimum of 3 h before fixing with 100 % methanol and staining with Giemsa–Wright solution (Fisher Scientific Cat. No. 123869). Slides were stained by immersion in eosinophilic dye $5\times 1\,\hbox {s}$ followed by $5\times {1}\,\hbox {s}$ in basophilic dye.

### Cholinesterase activity assay

Brain and muscle tissues were homogenized using a sonic dismembrator ($\hbox {Fisherbrand}^{\mathrm{TM}}$ Model 120 Sonic Dismembrator) 1:10 w:v with ice-cold 0.1 M potassium phosphate buffer (pH 7.2). Samples were centrifuged at 10,000 RPM in a Beckman Coulter microfuge 22R centrifuge held at ${4}\,^{\circ }\hbox {C}$ for 5 min. Total protein concentrations in tissue homogenates were determined by the Bradford assay (Sigma-Aldrich Cat. No. B6916). Cholinesterase activity was measured by the Ellman method adapted for a microplate reader (BioTek Synergy HT)^66^. Optimal assay conditions were 0.1 M potassium phosphate buffer (pH 7.2), 0.48 mM acetylcholine, 0.64 mM DTNB (Sigma-Aldrich Cat. No. D8130), 1.1 mM sodium bicarbonate. Assays were initiated through the addition of acetylcholine (Sigma-Aldrich, Cat. No. 01480) in a total volume of ${300}\,\upmu \hbox {L}$. Absorbance was read at 412 nm every 2.5 min for 10 min.

### Cloacal fluid

#### Collection

Cloacal fluid was collected for 1 h at 3 time points each day according to one of two schedules: starting (1) 1 h, 6 h, and 23 h, or (2) 2.5 h, 6.5 h, and 23 h after DT. Cloacal fluid was collected according to schedule (1) in pilot experiments, and (2) in the subsequent trials. A watch glass was placed beneath birds perching in 10 cm W $\times$ 12 cm H glass cylinder enclosures stopped with 19-gauge galvanized 1 cm hardware mesh openings in order to obtain cloacal fluid. To encourage greater cloacal fluid production, and to simulate the regular feeding behaviour of wild birds, individuals fed ad libitum from a syringe containing a 20% (w/v) sucrose solutions every 5 min to 10 min for the duration of cloacal fluid collection, which took place over 1 h as described under Sect. 4.2. After the collection period, cloacal fluid samples were stored at ${-20}\,^{\circ }\hbox {C}$ until pooling and refreezing prior to chemical analysis.

#### Chemical analyses

Cloacal fluid samples and dosing solutions were analyzed for IMI by HPLC-ESI-MS/MS by Laboratory Services, NWRC (National Wildlife Research Centre, Ottawa, ON, Canada). Cloacal fluid samples were pooled by time point across dosing days by individual to reach the necessary minimum volume of ${100}\,\upmu \hbox {L}$.

Cloacal fluid sample pools from 2018 trials were thawed at room temperature. Each pool was diluted 4 $\times$ with DI water (${25}\,\upmu \hbox {L}$ cloacal fluid + ${75}\,\upmu \hbox {L}$ DI water). The resulting ${100}\,\upmu \hbox {L}$ diluted samples were then spiked with ${100}\,\upmu \hbox {L}$ of internal standard (IS) solution. Spiked samples were filtered directly into ${300}\,\upmu \hbox {L}$ glass inserts using 4 mm PVDF ${0.45}\,\upmu \text {m}$ Millex filters and ${50}\,\upmu \hbox {L}$ aliquots were injected. For the 2018 analyses, the minimum detection limit (MDL) and minimum reporting limit (MRL) were ${0.204}\,\hbox {ng}\hbox { mL}^{-1}$ and ${0.616}\,\hbox {ng}\hbox { mL}^{-1}$ respectively.

Cloacal fluid sample pools from 2019 trials were thawed at room temperature and ${50}\,\upmu \hbox {L}$ of IS solution was added to $200\,\upmu \text {L}$ of pooled cloacal fluid. In cases where the sample volume was too small, volumes were adjusted: ${100}\,\upmu \hbox {L}$ cloacal fluid + ${25}\,\upmu \hbox {L}$ IS or ${80}\,\upmu \hbox {L}$ cloacal fluid + ${20}\,\upmu \hbox {L}$ IS as required. In these cases, duplicate injections of ${50}\,\upmu \hbox {L}$ were not possible. All samples were filtered with 4 mm PVDF ${0.45}\,\upmu \text {m}$ Millex filters prior to injection. For the 2019 analysis, the MDL and MRL were ${0.051}\,\hbox {ng}\hbox { mL}^{-1}$ and ${0.154}\,\hbox {ng}\hbox { mL}^{-1}$ respectively.

Cloacal fluid sample pools and dosing solutions were analyzed according to modifications to the methods of Main et al.^9^. Briefly, IMI in a cloacal fluid or DI water matrix was quantified by the internal standard method using the API5000 Triple Quadropole Mass Spectrometer (AB Sciex) and the TurboSpray ion source in positive polarity. The calibration curve was constructed from 8 concentrations ranging from ${0.1}\,\hbox {ng}\hbox { mL}^{-1}$ to ${20}\,\hbox {ng}\hbox { mL}^{-1}$ yielding an *R* greater than 0.995 (linear regression, no weighting). Injection cross-contamination was monitored by injecting solvent blanks (water:acetonitrile 80:20) before and after each set of samples. Contamination was also monitored by using a DI water sample blank spiked at ${20}\,\hbox {ng}\hbox { mL}^{-1}$ IMI. In all cases, no IMI above MDLs was detected. Method precision was evaluated by duplicate injections and/or duplicate dilutions: the RPDs (relative percent differences) were all less than 15 %, demonstrating good method precision. Method accuracy was evaluated by analyzing a ${20}\,\hbox {ng}\hbox { mL}^{-1}$ QC spike per set: the recoveries ranged between 96 % and 106 %, demonstrating good method accuracy.

### Statistical analyses

All statistical analyses were conducted in R version 3.5.2^67^. Birds exhibiting weight loss outside the lower bound of the 95 % confidence interval (CI) of 20 % over the study period ($n=3$) were omitted and data were reanalyzed. Birds exhibiting extreme weight loss across the pre-dose and post-dose conditions were in the control group ($n=2$) and the low dose group ($n=1$) suggesting a adverse response to the experimental period rather than the treatment itself. Data are presented as mean ± standard error. Significance ($p<0.05$) was determined in the DRC package by comparison of the fitted model to a simple linear regression with a slope of 0. In cases where a dose-response model was not a better fit to the data than a simple linear regression, as determined by the lack-of-fit test in the DRC package in R, a linear model was used. In cases where a linear model was used, omega-squared estimates of effect sizes and their confidence intervals where calculated using the effectsize package in R^68^.

#### Analyses and modeling of metabolic rate data

Instantaneous $\hbox {O}_2$ consumption rate (${\dot{V}}_{\hbox {O}_{2}}$, in $\hbox {mL}\cdot \hbox {O}_2\cdot \hbox {min}^{-1}$) and the respiratory exchange ratio of birds (RER; defined as the ratio between ${\dot{V}}_{\text {CO}_{2}}$ and ${\dot{V}}_{\text {O}_{2}}$) were determined across the 7 min dwell from chamber air sampled at 1 Hz with a flow rate of ${300}\,\hbox {mL min}^{-1}$, using standard equations. Data were then converted to a metabolic rate in $\hbox {J min}^{-1}$ by applying the following oxyjoule equivalency^69^:

1 $$\begin{aligned} MR=V_{{\hbox {O}_2}}\cdot (16+(5.164\cdot RER)) \end{aligned}$$

Instantaneous metabolic rate data (in $\hbox {J min}^{-1}$) were integrated over the duration of the dwell to calculate total energy expended during the dwell (in J). Assuming that hummingbirds were more likely to have been stressed immediately after handling and placement into respirometry chambers, we discarded first dwell data (collected within 30 min; Sect. 4.3) and included second dwells (collected between 30 min to 60 min; Sect. 4.3) for comparison only. Energy expenditure for each bird was normalized by dividing mean energy expenditure across post-dose days by mean energy expenditure across pre-dose days. Normalized mean energy expenditure in J was modeled across dosing groups by an asymmetric, alternative parameterization of the 3 parameter Weibull dose-response model (Weibull type 2)^70,71^. The model was fitted using the DRC package with the general form^71^:

2 $$\begin{aligned} f(x) = a\cdot e^{\left( -e^{\left( b\cdot \log (x)-c\right) }\right) } \end{aligned}$$

where it is assumed that $\lim _{x\rightarrow \infty } f(x)=0$. The biological interpretation of this theoretical limit assumes that as the concentration of IMI increases indefinitely, the metabolic rates of the birds would approach 0, as they would be dead. Parameter *a* characterizes the mean energy expenditure of birds unexposed to IMI (dose$= 0$). Parameter *b* relates to the LC50 and characterizes location and steepness of the upper shoulder of the dose-response. The sigmoidal model is asymmetric about the inflection point characterized by parameter *c*. Significance ($p<0.05$) was determined in the DRC package by comparison of the fitted model to a simple linear regression with a slope of 0, indicating no effect of dose on energy expenditure.

#### Analyses of behavioural assay

The number of flight instances, amount of time feeding, and whether feeding occurred during a flight instance were processed from each 2 h set of videos. The change in mean time in flight on each day for each individual between pre-dose and post-dose conditions was calculated for foraging and non-foraging flights. Linear models of foraging and non-foraging flight time as a function of dose were analyzed by one-way ANOVA, with an alpha level set at 0.05. The total number of foraging and non-foraging flight, average consumption of maintenance diet per instance of flight where birds engaged in feeding, and average duration of foraging and non-foraging flight were also compared among groups in this manner.

#### Analyses of heterophil/lymphocyte ratios

Manual 100-cell differential leukocyte counts were conducted on smears under $1000\times$ magnification. Fields of view were excluded from differential counts if cells did not form a monolayer or if thrombocyte aggregates were present. Differential leukocyte counts of blood smears were duplicated for each slide by 2 individuals where heterophils, eosinophils, monocytes and lymphocytes were tallied up to 100. Both individuals were blind to the dose groupings. The ratio of heterophils to lymphocytes was then calculated and the mean of the two duplicate ratios was used for analyses. Intraclass correlations for duplicate readings was calculated at 0.75 with upper and lower 95% confidence bounds of 0.90 and 0.45 using the DescTools package^72^. A linear model of heterophil/lymphocyte ratios as a function of dose were analyzed by one-way ANOVA, with an alpha level set at 0.05.

#### Analyses of cholinesterase activity

The specific activity of cholinesterase was modeled by linear regression in brain and muscle tissues. $\Delta$ (difference in) absorbance values were calculated from absorbance 5 min to 10 min after the reaction was initiated. Linear models of cholinesterase activity as a function of dose were analyzed by one-way ANOVA, with an alpha level set at 0.05.

#### Toxicokinetic modeling

For the general first-order toxicokinetic excretion model,

3 $$\begin{aligned} {[}U] = a\cdot e^{-k\cdot x} \end{aligned}$$

where [*U*] is the concentration of IMI in cloacal fluid at time *x* in hours, model parameters *a* and *k* represent the excretion coefficient and elimination rate constant of unmetabolized IMI in cloacal fluid, respectively. Convergence on parameter estimates was achieved by nonlinear least-squares regression, and 95% CIs for excretion models were determined by bootstrap resampling in the nlstools package^45^. Elimination half-life of imidacloprid was calculated according to the following equation:

4 $$\begin{aligned} t_{1/2} = \frac{\ln 2}{k} \end{aligned}$$

## Supplementary information

Supplementary Information.
