# Supplementary material for: Neonicotinoid pesticides exert metabolic effects on avian pollinators
Source: Sci Rep. 2021 Feb 3;11:2914. doi: 10.1038/s41598-021-82470-3 (PMC7858574; doi:10.1038/s41598-021-82470-3)
Supplement: Supplementary file 1 — Supplementary Information. [file 41598_2021_82470_MOESM1_ESM.pdf]

Neonicotinoid pesticides exert metabolic effects on avian pollinators

Simon G. English[1,2], Natalia I. Sandoval-Herrera[2,3], Christine A. Bishop[4], Melissa Cartwright[2], France Maisonneuve[5], John E. Elliott[4], and Kenneth C. Welch Jr.[*,1,2,3,6]

[1] *Department of Cell & Systems Biology, University of Toronto*
[2] *Department of Biological Sciences, University of Toronto Scarborough*
[3] *Department of Ecology & Evolutionary Biology, University of Toronto, Scarborough*
[4] *Wildlife Research Division; Environment and Climate Change Canada*
[5] *Ecotoxicology and Wildlife Health Division; Environment and Climate Change Canada*
[6] *Centre for the Neurobiology of Stress, University of Toronto Scarborough*
[*] *kenneth.welchjr@utoronto.ca*

1 Supplemental figures

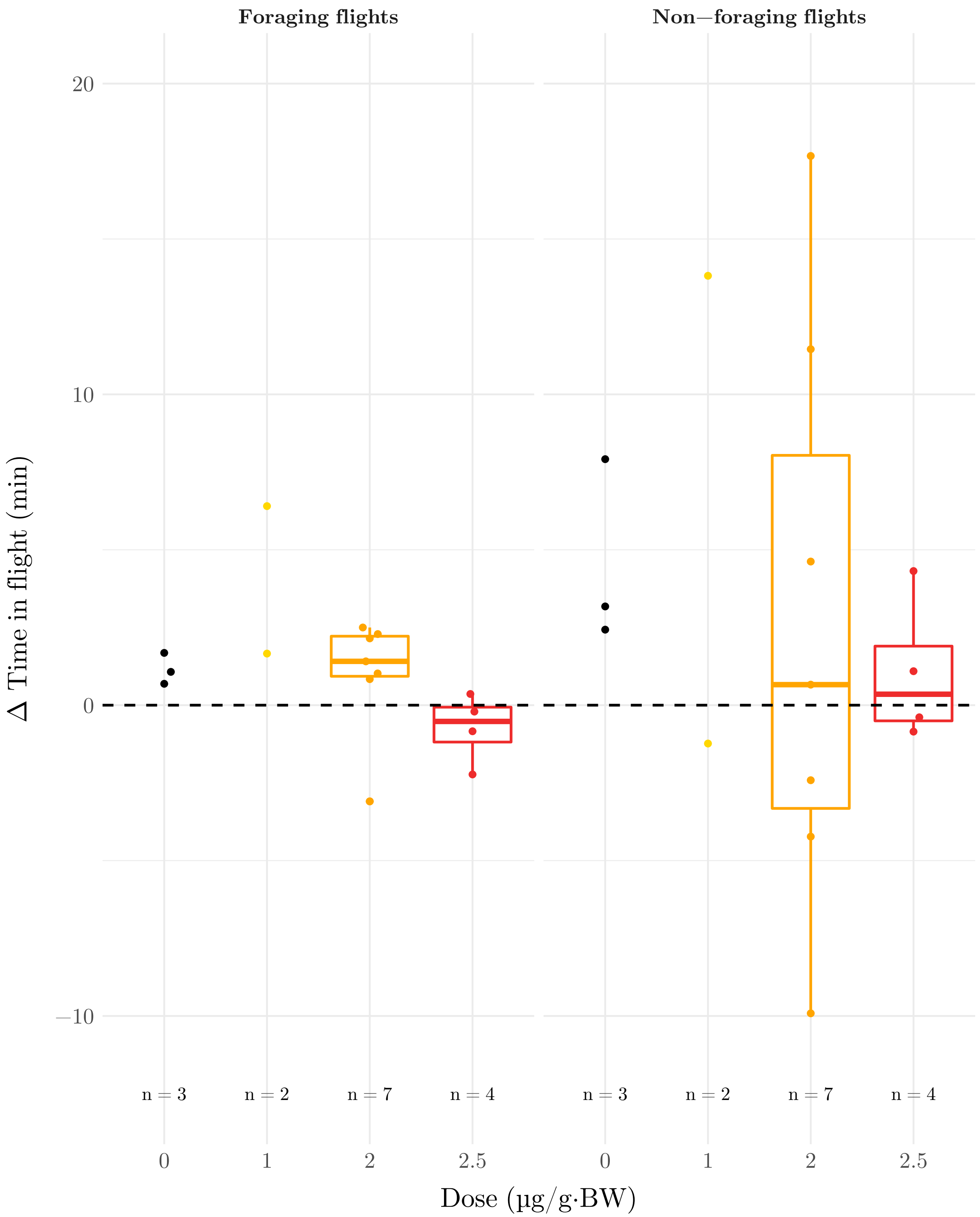

Figure S1. Change in time spent in flight for birds in post-dose group relative to individual birds' pre-dose time spent in flight. Plots are faceted by foraging and non-foraging flights. No significant effect of dosage was observed on time spent in foraging flights or non-foraging flights among dosing groups ($p > 0.05$). Effect sizes for change in time spent in foraging flights was large (0.29) but small for non-foraging flights (−0.16). Values above, and below, the dashed line indicate a positive, or negative, change in time spent flying between pre-dosing and post-dosing conditions, respectively.

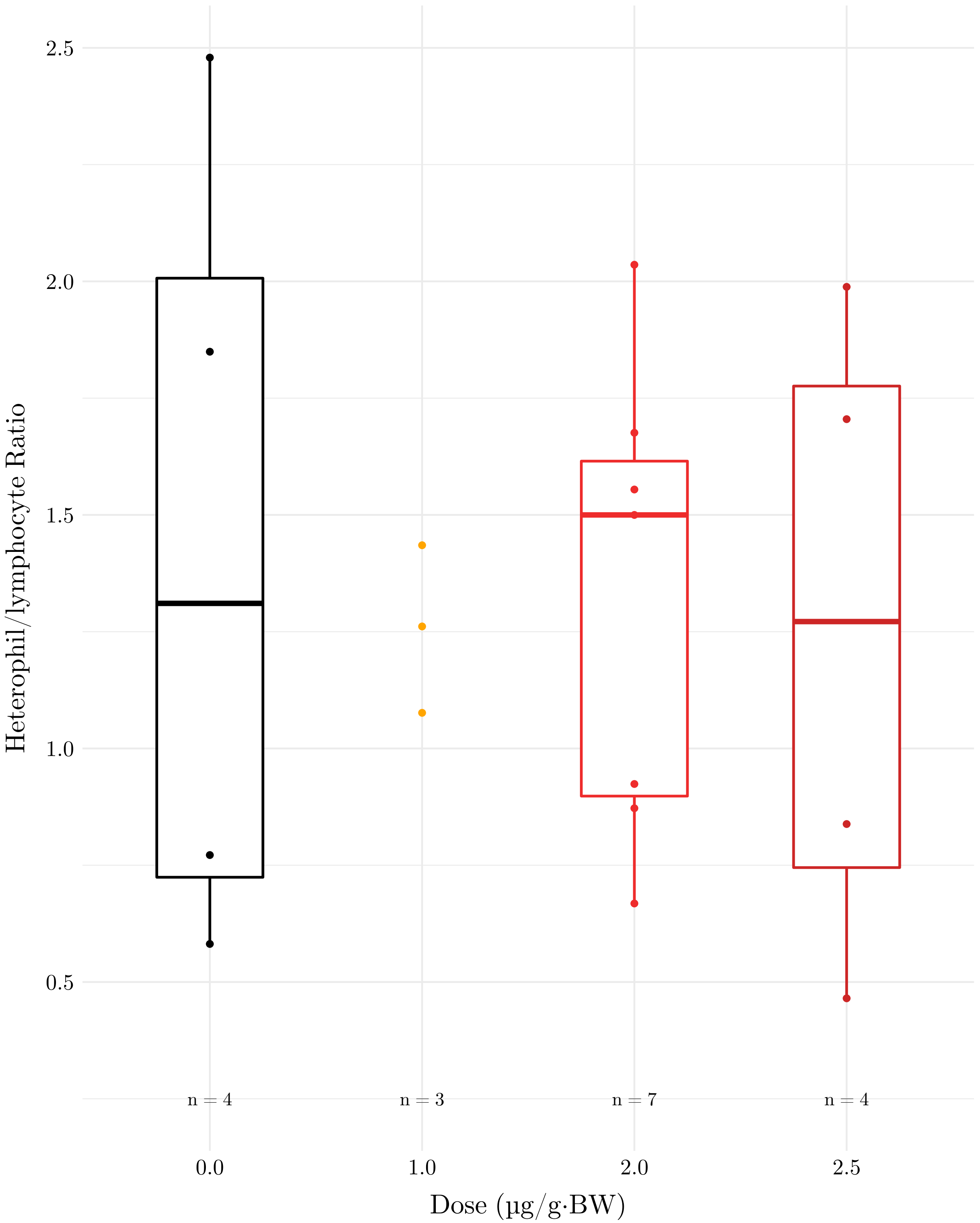

Figure S2. Heterophil/lymphocyte ratios of ruby-throated hummingbirds exposed to three acute doses of imidacloprid ranging 0.0 µg g^{-1} to 2.5 µg g^{-1}·Body Weight (BW). No significant effect of acute dosing was observed on heterophil/lymphocyte ratios ($p > 0.05$; effect size: -0.19).

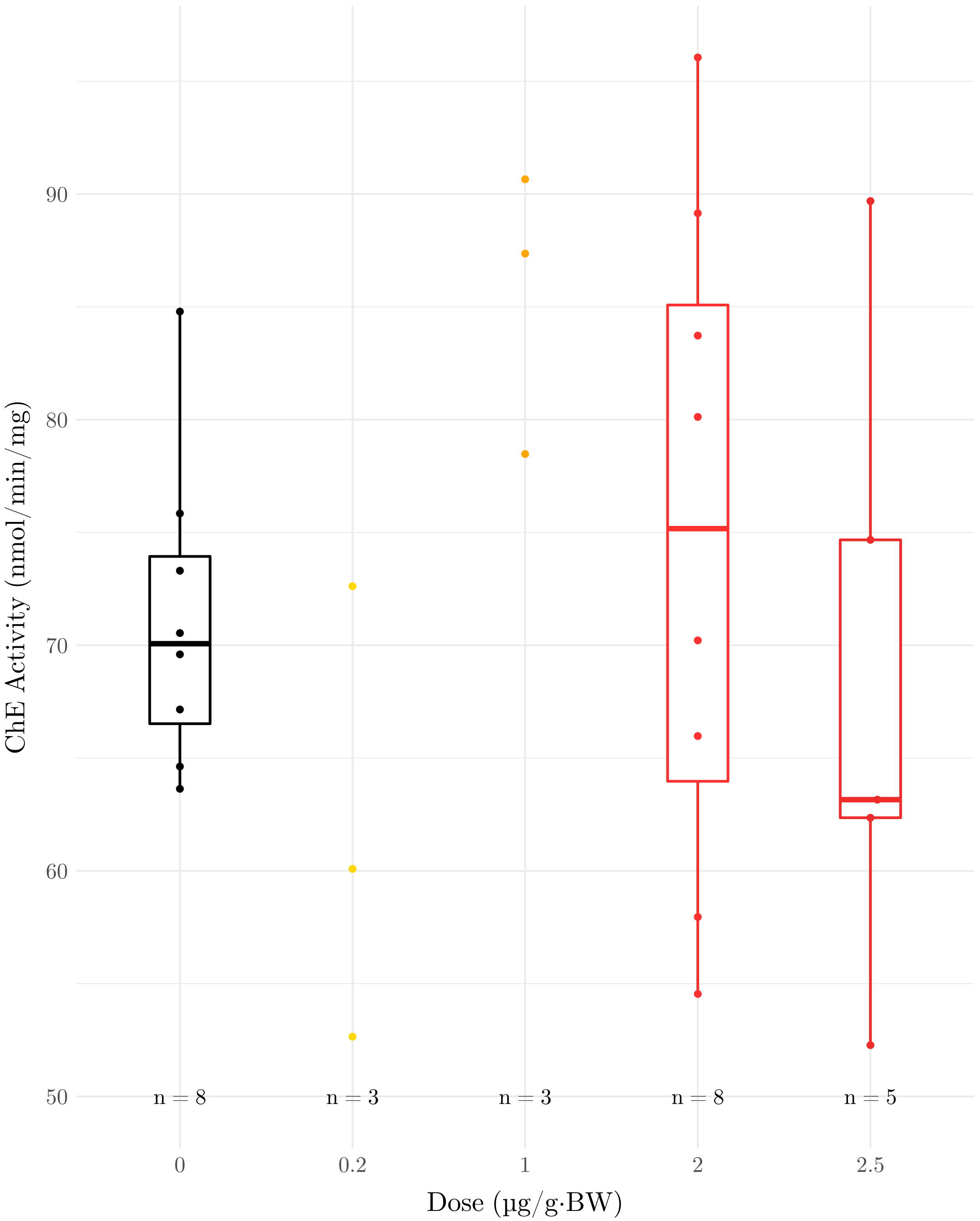

Figure S3. Specific activity of cholinesterase enzymes in brain tissue of ruby-throated hummingbird orally dosed with imidacloprid 0.0 µg g^{-1} to 2.5 µg g^{-1}·Body Weight (BW). No significant differences in enzyme activity per mg total protein were observed between dosing groups ($p > 0.05$; effect size: 0.11).

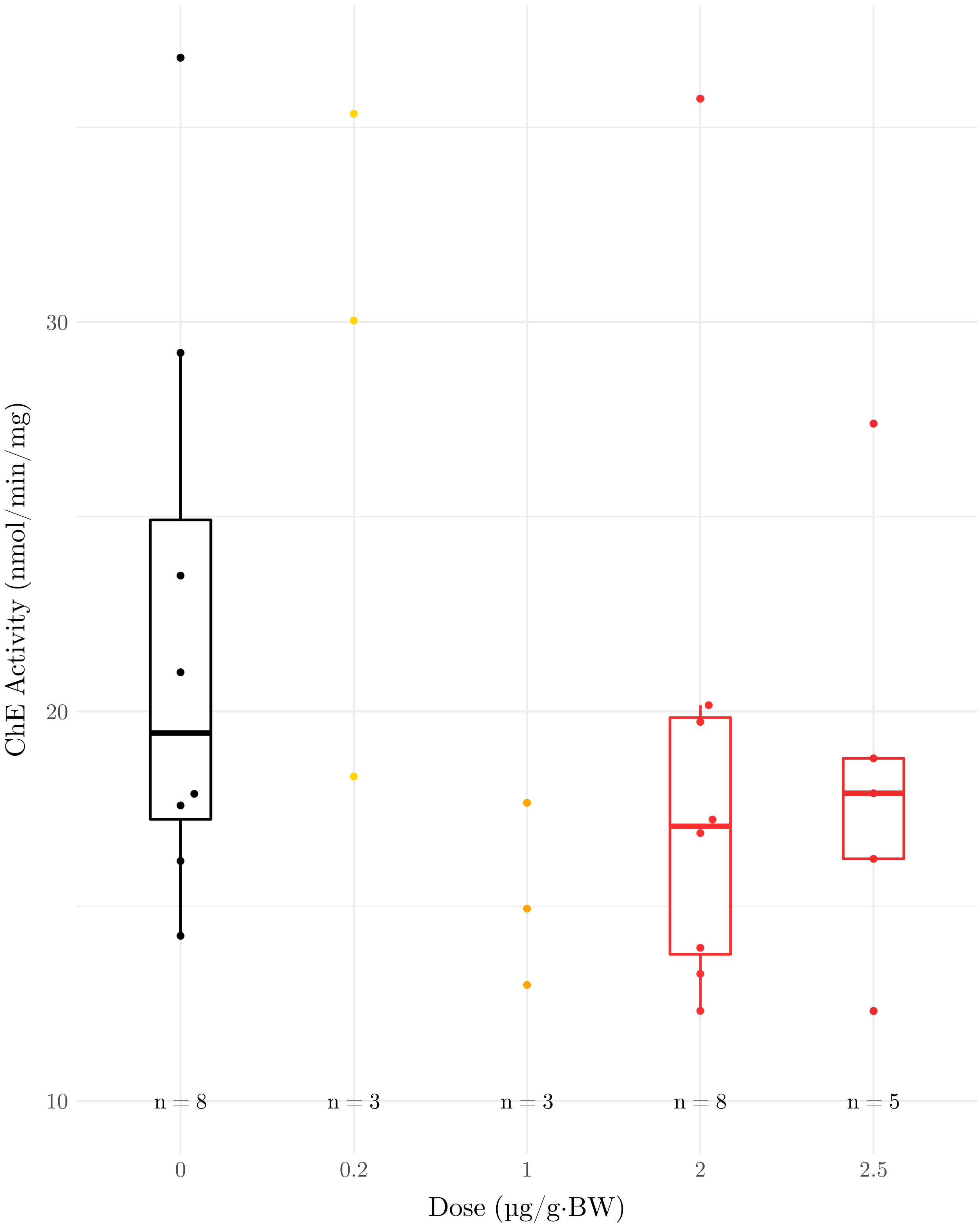

Figure S4. Specific activity of cholinesterase enzymes in muscle tissue of ruby-throated hummingbirds orally dosed with imidacloprid 0.0 $\mu g\, g^{-1}$ to 2.5 $\mu g\, g^{-1}$·Body Weight (BW). No significant differences in enzyme activity per mg total protein were observed between dosing groups ($p > 0.05$; effect size: 0.08).

2 Supplemental data

2.1 Mass and maintenance diet intake

Dosing group did not significantly affect percent weight lost during the experimental period, though birds across all conditions lost 13 % ± 3 % of their body mass on average (**Table S1**). Birds in breeding seasonality had significantly lower average body masses over the experimental period than birds in wintering/migratory seasonality, as determined by a two-tailed t-test where group means were 2.83 g ± 0.03 g and 3.47 g ± 0.11 g respectively (p<0.001). Additionally, average daily consumption of their maintenance diet during the experimental period was significantly higher among birds in breeding seasonality than birds in wintering/migratory seasonality, as determined by a two-tailed t-test where group averages were 10.3 mL ± 0.3 mL and 7.8 mL ± 0.4 mL respectively (p<0.001).

Table S1. Proportion of mass lost from first to last day of experimentation, grouped by dose.

			95% CI	
Dose ($\mu g\,g^{-1}\cdot$BW)	Estimate	Std. Err.	Lower	Upper
(Intercept)	0.87	0.03	0.80	0.93
0.2	0.02	0.07	-0.12	0.15
1.0	0.01	0.06	-0.11	0.13
2.0	0.04	0.05	-0.05	0.14
2.5	0.06	0.05	-0.05	0.17

Average intake of maintenance diet on post-dose days normalized to pre-dose daily averages did not differ significantly between dosing groups (95% CI: -0.12–0.05). Intake of maintenance diet on post-dose days normalized to pre-dose daily averages did not differ significantly between dosing groups during each cloacal fluid collection (Section 4.7.1) or in the first 30 min immediately after dosage (i) 0 h; 95% CI: -0.42–0.14, ii) 2.5 h; 95% CI: -0.25–0.01, iii) 6.5 h; 95% CI: -0.14–0.07, iv) 23 h; 95% CI: -0.27–0.06).

2.2 Considerations of mass loss in hummingbirds

Effects of imidacloprid exposure on body mass have been observed in white-crowned sparrows (*Zonotrichia leucophrys*)[33]; however, we did not observe similar trends attributable to dosing with imidacloprid (**Table S1**). The experimental intervention, which included a period of imposed fasting for respirometry experiments, induced an average decline in body mass of 13 % ± 3 % across all dosing groups. Individual hummingbirds' masses are subject to considerable variation over relatively short timescales, and future experiments that aim to test the effects of pesticide exposure should consider the impact of chronic low doses of pesticides on birds' masses over longer timescales than those used in this study.

Table S2. Summary statistics of cloacal fluid analytical chemistry results. All times are relative to dosing. Doses are reported in $\mu g\, g^{-1}$·Body Weight (BW). Maximum, minimum, and mean values are reported in $ng\, mL^{-1}$. Samples were pooled by time point across dosing days by individual. One control bird showed higher than non-detectable levels at each time point in the pre-dosing sample pool.

Dose	Time (h)	Max	Min	Mean	n
0.0	2.50	0.14	0.00	0.02	7
0.0	6.50	0.13	0.00	0.02	6
0.0	23.00	0.32	0.00	0.05	7
0.2	1.00	555.00	310.00	410.33	3
0.2	6.00	144.00	67.50	99.00	3
0.2	24.00	41.30	34.60	38.87	3
1.0	2.50	1460.00	820.00	1050.50	4
1.0	6.50	424.00	161.00	290.75	4
1.0	23.00	43.10	11.20	21.75	4
2.0	2.50	2010.00	909.00	1328.62	8
2.0	6.50	542.00	160.00	360.12	8
2.0	23.00	43.50	6.05	20.53	8
2.5	1.00	4520.00	3570.00	4076.67	3
2.5	2.50	2390.00	1810.00	2050.00	4
2.5	6.00	740.00	370.00	600.00	3
2.5	6.50	766.00	475.00	645.00	4
2.5	23.00	41.80	18.00	30.95	4
2.5	24.00	90.00	69.50	79.50	3
